# Supplementary material for: Biodegradation of Selected Hydrocarbons by Fusarium Species Isolated from Contaminated Soil Samples in Riyadh, Saudi Arabia
Source: J Fungi (Basel). 2023 Feb 6;9(2):216. doi: 10.3390/jof9020216 (PMC9966121; doi:10.3390/jof9020216)
Supplement: Supplementary file 1 [file jof-09-00216-s001.zip › jof-2161801-Supplementary.pdf]

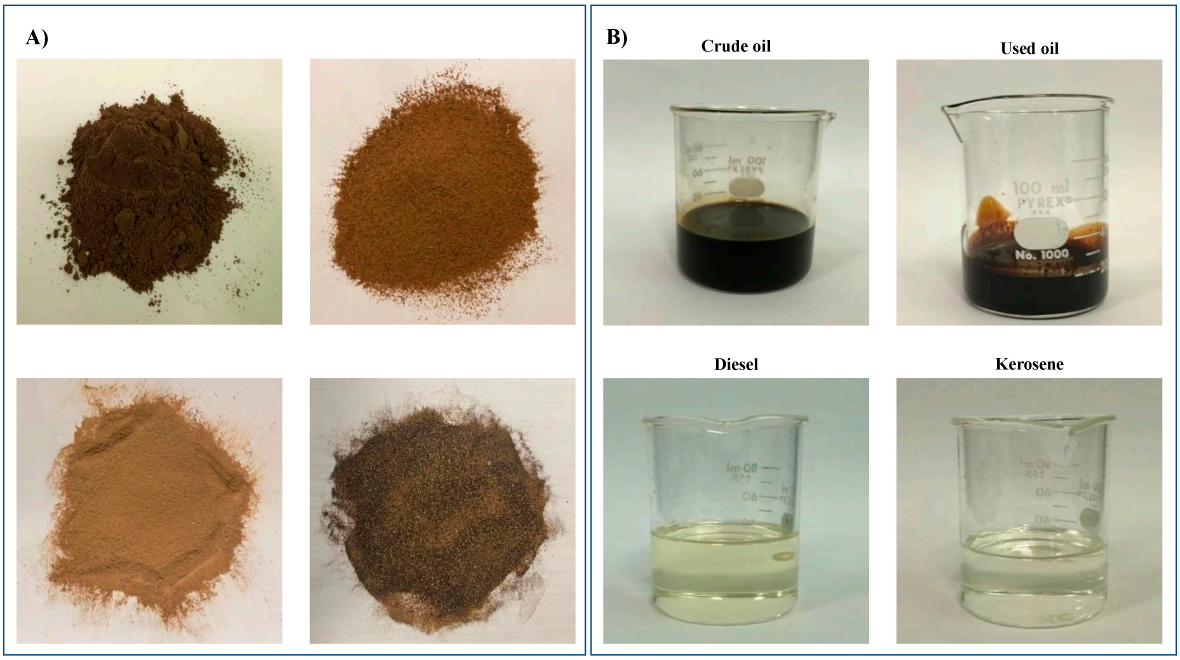

Supplementary Figure S1. soil and hydrocarbon samples used in the current study, A) Soil Samples were collected from contaminated soil-samples in oil-reservoirs from Riyadh, Saudi Arabia, B) the oil-hydrocarbons were collected from oil-pipelines of Aramco Company, Al-Dammam, Saudi Arabia. www.mdpi.com/xxx/s2,.

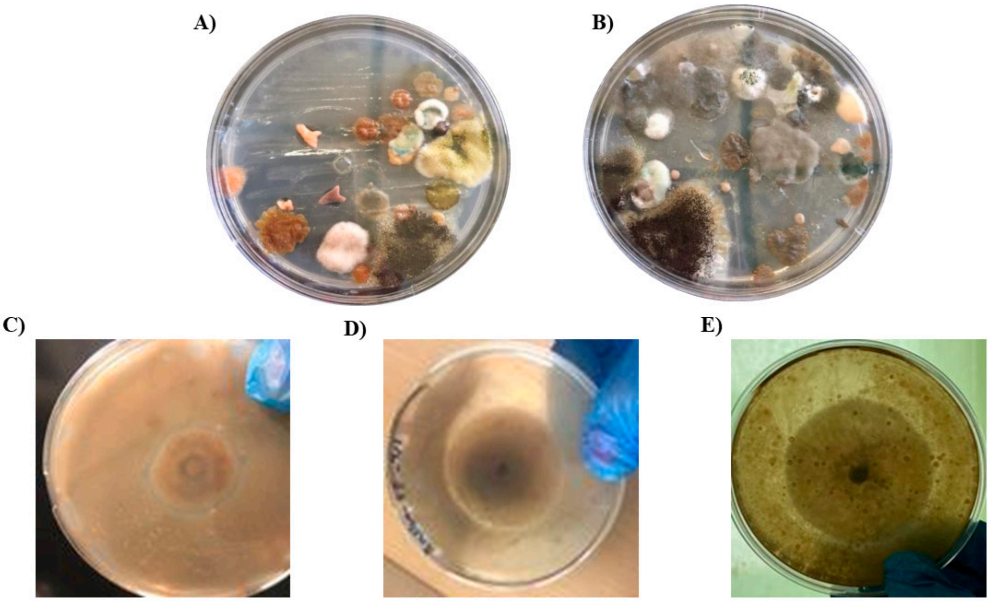

Supplementary Figure S2. Culture of the fungi were investigated in the contaminated soil samples cultured on PDA Petri dishes A) and B). Separated colonies growing in MSM medium C) *F. verticillioides,* D) *F. oxysporum*, and E) *F. proliferatum* www.mdpi.com/xxx/s3,.

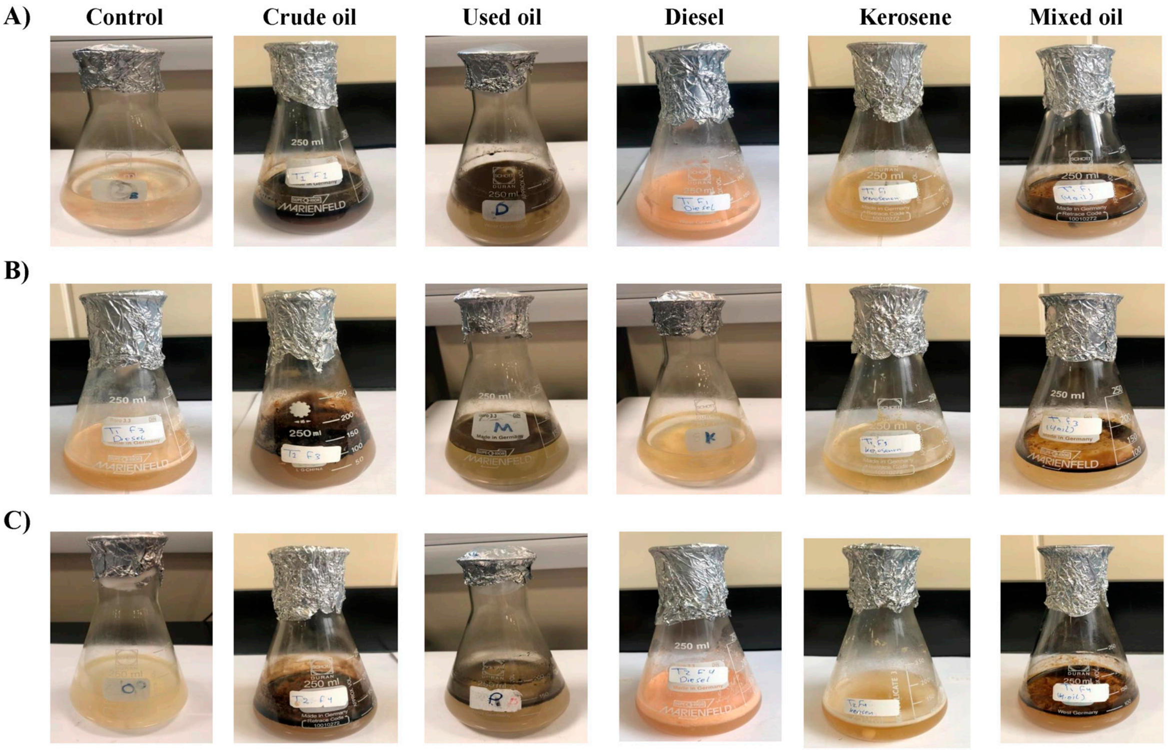

Supplementary Figure S3. Biodegradation ability of isolated fungi cultured on Liquid MSM media supplied with 1% of various oil sources and incubated in a shaker at 25°C for 30 days. A) *F. verticillioides,* B) *F. proliferatum*, C) *F. oxysporum*. www.mdpi.com/xxx/s4,.

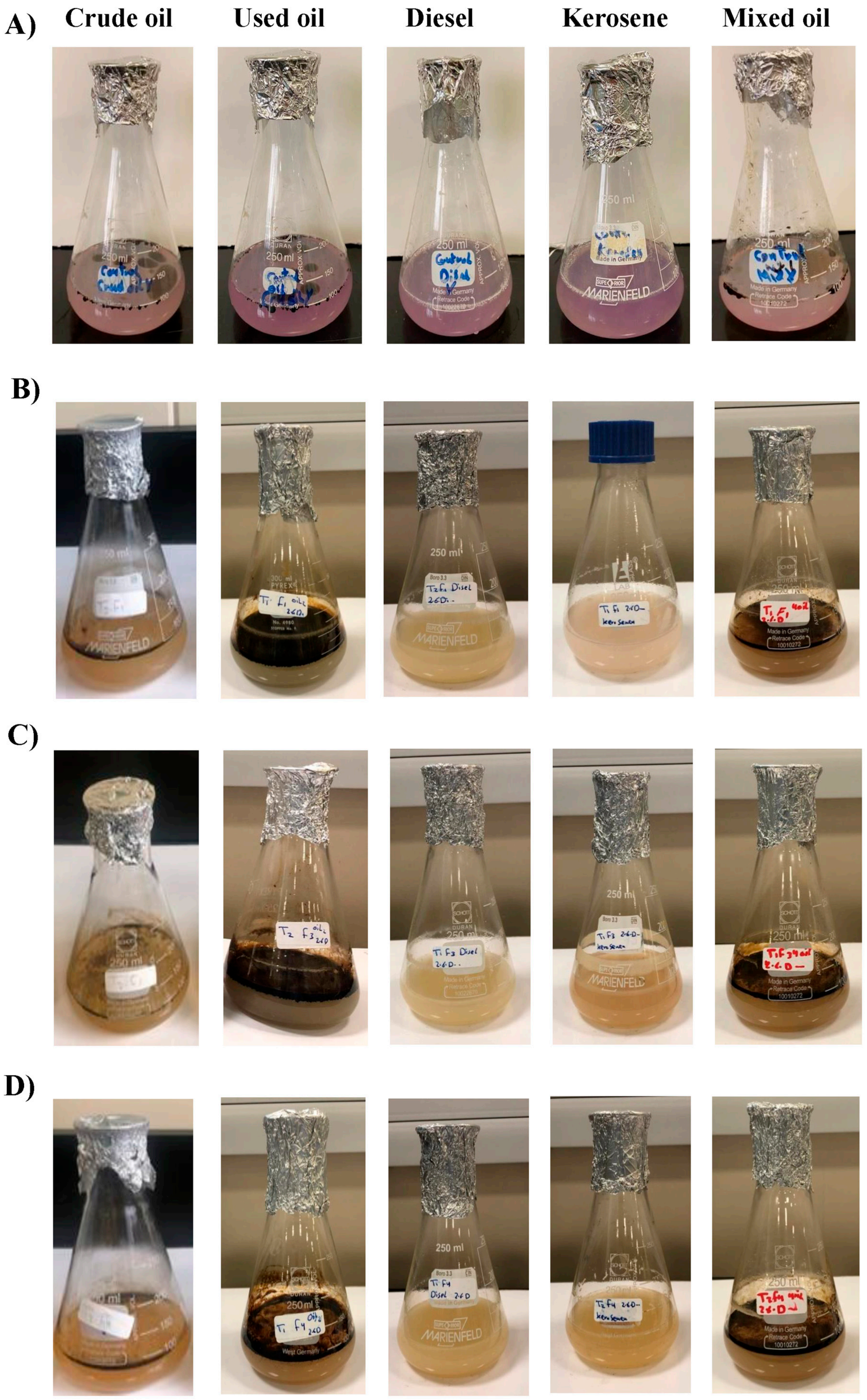
A)
Crude oil
Used oil
Diesel
Kerosene
Mixed oil
B)
C)
D)

Supplementary Figure S4. DCPIP assay. Liquid media (MSM) containing 1% of various oil sources; 0.1% (v/v) of Tween 80, 0.6 mg/mL of redox indicator (DCPIP) as a control and incubated in a shaker at 25°C for 15 days. A) control (no fungi), B) *F. verticillioides*, C) *F. proliferatum*, D) *F. oxysporum*. www.mdpi.com/xxx/s5,.

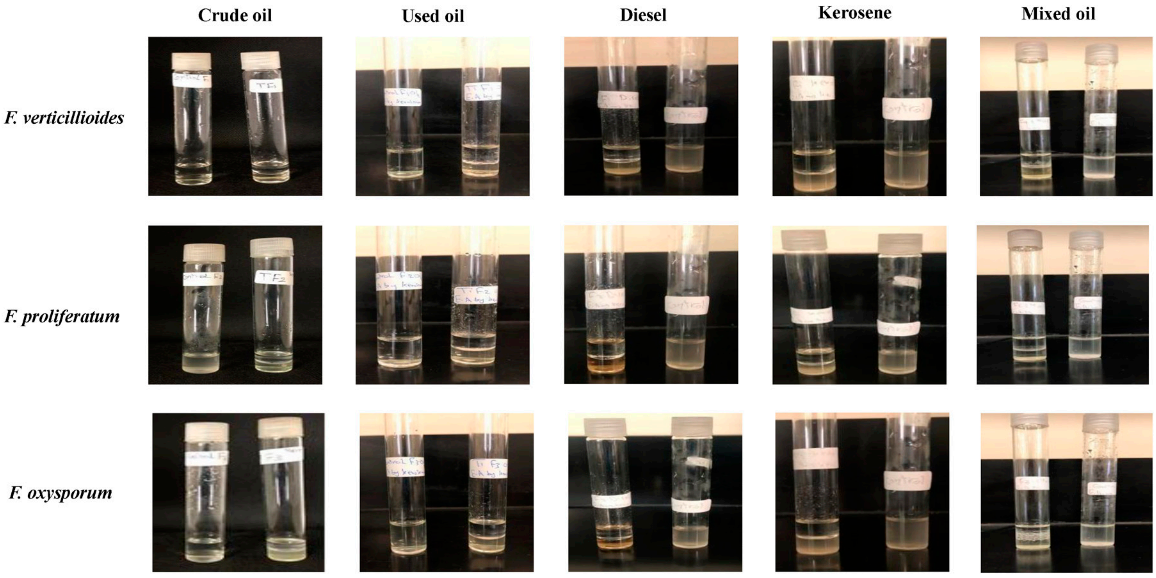

Supplementary Figure S5. Emulsification test. The emulsification activity was measured to test the ability of the CFSs produced by different *Fusarium* isolates to emulsify different hydrocarbons.
